# Physiological and Morphological Responses of Okra (*Abelmoschus esculentus* L.) to *Rhizoglomus irregulare* Inoculation under Ample Water and Drought Stress Conditions Are Cultivar Dependent

**DOI:** 10.3390/plants11010089

**Published:** 2021-12-28

**Authors:** Amna Eltigani, Anja Müller, Benard Ngwene, Eckhard George

**Affiliations:** 1 Leibniz Institute of Vegetable and Ornamental Crops (IGZ), Theodor-Echtermeyer-Weg 1, 14979 Großbeeren, Germany; mueller@igzev.de (A.M.); Benard.Ngwene@agcocorp.com (B.N.); george@igzev.de (E.G.); 2 Department of Crop and Animal Sciences, Plant Nutrition, Humboldt University, Unter den Linden 6, 10099 Berlin, Germany; 3 AGCO South Africa (Pty) Ltd., 16 Pomona Road, Pomomna, Kempton Park 1619, South Africa

**Keywords:** AMF, drought, okra, cultivar, P uptake, mycorrhizal dependency, root morphology, breeding background

## Abstract

Okra is an important crop species for smallholder farmers in many tropical and subtropical regions of the world. Its interaction with mycorrhiza has been rarely studied, and little is known about its mycorrhizal dependency, especially under drought stress. In a glasshouse experiment, we investigated the effect of Arbuscular Mycorrhiza Fungi (AMF) inoculation on growth, evapotranspiration, mineral nutrition and root morphology of five okra cultivars under ample water and drought stress conditions. ‘Khartoumia’, ‘HSD6719’, ‘HSD7058’, ‘Sarah’ and ‘Clemson Spineless’-cultivars commonly used by farmers in Sudan were chosen for their geographical, morphological and breeding background variations. The plants were either inoculated with *R. irregulare* or mock-inoculated. Seven weeks after seeding, the soil–water content was either maintained at 20% *w*/*w* or reduced to 10% *w*/*w* to impose drought stress. Drought stress resulted in plant P deficiency and decreased shoot dry biomass (DB), especially in HSD7058 and Clemson Spineless (69% and 56% decrease in shoot DB, in the respective cultivars). Plant inoculation with AMF greatly enhanced the shoot total content of P and the total DB in all treatments. The mycorrhizal dependency (MD)—the degree of total plant DB change associated with AM colonization—differed among the cultivars, irrespective of the irrigation treatment. Key determinants of MD were the root phenotype traits. Khartoumia (with the highest MD) had the lowest root DB, root-to-shoot ratio, and specific root length (SRL). Meanwhile, HSD6719 (with the lowest MD) had the highest respective root traits. Moreover, our data suggest a relationship between breeding background and MD. The improved cultivar Khartoumia showed the highest MD compared with the wild-type Sarah and the HSD7058 and HSD6719 landraces (higher MD by 46%, 17% and 32%, respectively). Interestingly, the drought-affected HSD7058 and Clemson Spineless exhibited higher MD (by 27% and 15%, respectively) under water-deficiency compared to ample water conditions. In conclusion, the mediation of drought stress in the okra plant species by AMF inoculation is cultivar dependent. The presence of AMF propagules in the field soil might be important for increasing yield production of high MD and drought susceptible cultivars, especially under drought/low P environments.

## 1. Introduction

Drought is one of the most widespread problems in agriculture. It severely reduces plant biomass, leading to diminished crop productivity [1,2,3]. Under drought stress conditions, not only water but also nutrient availability may be severely decreased compared to ample water conditions [4]. Low soil moisture can result in disturbed nutrient diffusion and mass flow, and drought therefore often limits the availability of nutrients, especially P [5,6].

The plant root is the first organ to encounter soil moisture changes. Thus, root morphological and physiological responses to drought stress are important adaptations [7]. Substantial variations in root morphology (root length, surface area, volume and diameter) have been reported among plant species and within genotypes of plant species [8,9,10,11,12]. Variations in root morphological traits, such as length and diameter, have been used to classify soybean cultivars into different root phenotypes that could be important in conferring plant biomass production under drought [13]. Moreover, under limited soil moisture conditions, plants often re-allocate assimilates to participate in root rather than shoot growth, thereby increasing root extension into deeper soil layers [14]. 

Mycorrhizal dependency (MD) is the degree of plant dry biomass (shoot or total dry biomass) change associated with arbuscular mycorrhizal fungi (AMF) colonization [15]. According to Bowles et al. [16], MD is calculated based on the plant dry biomass (shoot or total ) using the individual values of the dry biomass of AM—inoculated plants and mean values of the dry biomass of non-inoculated plants within each treatment. Variation in MD has been frequently observed among different cultivars of many plant species, such as corn, rice, maize, soybean, chickpea, cowpea and sorghum [17,18,19,20,21,22,23]. A correlation between the root morphological traits and the MD was reported by Tawaraya [15]. The author reported that root traits (diameter, hair length and hair density) might be direct determinants for MD. Moreover, MD differed between geographically diverse *Medicago truncatula* accessions [24,25], which had diverse phenotypes in plant shoot biomass and root length that were linked to the conditions in their geographic-specific environment, including climate and soil characteristics. From an AM evolutionary point of view, Hetrick et al. [26] observed that wheat cultivars released before 1950 displayed more MD than those released later. He suggested that germplasm selection under high phosphorus fertilizer conditions could have reduced the frequency of genes for the mycorrhizal dependence of wheat. On the other hand, other studies showed no adverse effects on mycorrhizal growth response (plant shoot dry biomass) owing to modern breeding programs [27].

Although AMF occur naturally in almost all agricultural fields, their occurrence and diversity may be reduced following the application of unsuitable agricultural cultural practices such as tillage, monoculture, choice of crop and other cropping practices [28]. Management of AMF in agricultural fields could be a promising tool for enhancing crop productivity especially under limited soil moisture and/or nutrient availability.

Okra (*Abelmoschus esculentus* L. Moench.) is widely grown in tropical and subtropical regions including Sudan, where it is the most commonly grown vegetable and the staple food of the Sudanese. A genotype ‘Sarah’, representing the wild-type of okra is mainly used as a dry powder in food preparation, while the more domesticated okra types are mainly used fresh. The large genotypic variability in Sudan comprises rain-fed and irrigated types of okra [29]. This includes the cultivars ‘Khartoumia’, ‘HSD6719’, ‘HSD7058’, ‘Clemson spineless’, and the wild-type ‘Sarah’. Khartoumia’, a locally improved cultivar, is the cultivar most frequently grown in the irrigated sector in the Khartoum area. ‘HSD6719’ is a farmer cultivar (landrace) grown in El Fuda, South Kordofan state, Sudan. The area is characterized by a hot semi-arid climate with a unimodal rainfall pattern (760 mm per year). The soil is characterized by low P (<15 ppm and reaching 7 ppm in some sites). Vegetables including okra are grown under unreliable rainfall conditions and low agrochemical inputs. ‘HSD7058’ is a farmer cultivar (landrace) grown in Arbaat, El Gonab, Red Sea State, Sudan. The Arbaat area constitutes a khor (wadi, valley or ravine), bounded by relatively steep banks, which in the rainy season becomes a watercourse. Although rainfall is insufficient to support rain-fed cropping in the area, water from the khor is harvested and used for garden crop irrigation on the relatively fertile sedimentary soil. The farmer cultivars are usually selected by farmers during harvest based on morphological characteristics, such as the size of the plant, color or size of the fruits. The seeds of the healthy plants are reserved for growing the next crop. ‘Sarah’ is a wild-type okra and grows in different rain-fed parts of Sudan. ‘Clemson Spineless’ is an introduced cultivar (selected by Clemson University, USA) grown in the irrigated sector.

In Sudan, okra is grown mainly by subsistence farmers under low input of agrochemicals or irrigation. Despite its ecological and economic importance and role in human nutrition, mycorrhiza research has paid very little attention to okra up to now, concentrating instead on the few plant species that have global industrial importance. Little is known about AMF effects on root morphology and nutrient uptake in okra plants under drought stress conditions. Moreover, cultivar differences in okra response to mycorrhizal colonization have not been addressed. 

Here, we investigate the effect of AMF inoculation on the shoot and root dry biomass, root development, evapotranspiration and the content of P, N, Fe and Zn in the shoot of five okra cultivars under ample water and drought stress conditions. The cultivars ‘Khartoumia’, ‘HSD6719’, ‘HSD7058’, and ‘Clemson spineless’, commonly grown by Sudanese farmers in different regions, and the wild-type ‘Sarah’ were selected for their geographical, morphological and genetic (breeding) variations.

## 2. Materials and Methods

### 2.1. Experimental Design and Growth Conditions

The experiment was set up with a factorial design with 3 factors: (1) AMF inoculation treatment, comprising non-inoculated (−M) and inoculated plants (+M); (2) the okra cultivars ‘Khartoumia’, HSD6719, ‘HSD7058’, ‘Sarah’ and ‘Clemson Spineless’; and (3) a water regime, comprising ample water conditions (+W) and drought stress conditions (−W). Four replicates were used for each treatment, giving a total of 80 plants. The experiment was conducted in a glasshouse at the Institute of Vegetable and Ornamental Crops (IGZ) in Grossbeeren, Germany (52°22′ N, 13°20′ E). Plants were cultivated from June to August and randomly placed on tables in the glasshouse where the average day/night temperatures were 26 °C/22 °C and relative air humidity averaged 62%. Air humidity in Sudan ranges between 22% and 73%. It is worth mentioning that low air humidity in the desert climate and semi-arid climate in Sudan exist especially outside of the monsoon season. This is an important abiotic factor to consider besides the soil moisture factor when translating the results of this study to field conditions. 

### 2.2. Growth Substrate and AM Fungal Inoculation

Eighty black, round 2.5 L plastic pots (Teku Container BC 17; Pöppelmann, Germany) were filled with 2540 g of growth substrate. To obtain a growth substrate suitable for this experiment, material from the C-horizon of a Luvisol (loamy sand) was sieved and mixed 1:1 (*v*/*v*) with quartz sand. The loamy sand substrate was chosen because of its poor available P content. Calcareous soils, such as the substrate used here, have a strong tendency to fix P [5]. Therefore, the substrate used in the present work was chosen to reflect the soil conditions in arid areas, such as Sudan, where soils are often calcareous and have high pH. We mixed the loamy sand substrate with the quartz sand to allow easier separation and washing of the root system from the substrate at harvest time. The substrate mixture was characterized as 72% sand, 21% silt and 7% clay. 

Prior to use in the experiment, the substrate was dry-heated (85 °C/48 h) to eliminate any existing AMF propagules. Thereafter, the mineral nutrients were added to the substrate as a nutrient solution. The following amount of salts were added to the substrate: 200 mg N (NH_4_NO_3_), 50 mg P (KH_2_PO_4_), 200 mg K (K_2_SO_4_), 100 mg Mg (MgSO_4_), 10 mg Cu (CuSO_4_), 10 mg Fe (Fe-EDTA) and 10 mg Zn (ZnSO_4_) kg^−1^ dry substrate. The salts were solubilized in deionized water and then carefully mixed into the substrate. The fertilized substrate was incubated at room temperature before it was used in the experiment. The aim of this fertilization was to provide the plants with an optimal supply of all nutrients except for P (low availability in the substrate). This nutrient supply condition prevails in many Sudanese agricultural fields. The chemical properties of the mixture substrate after dry heating are described in Table A1. 

*Rhizoglomus irregulare* inoculum was produced in a glasshouse at IGZ, Grossbeeren, Germany. The starter inoculum was quartz–sand-based and contained propagules of *Rhizoglomus irregulare* supplied by INOQ GmbH (Schnega, Germany). To establish AMF treatment (+M), the fungal inoculum (air-dried AM colonized root pieces and adhering substrate containing extraradical AM mycelium with spores, a substrate similar to the one used in the experiment) was homogeneously mixed with the growth substrate at a rate of 7% (*w*/*w*). The non-mycorrhizal plants (−M) were inoculated with the same amount of dry heated inoculum plus a filtrate of non-dry-heated (oven at 85 °C for 48 h) mycorrhizal inoculum to obtain the same initial microflora and soil physical properties across all treatments. Six weeks after the sowing date, plant roots were checked to confirm AMF colonization. To determine the AMF-colonized root length, samples of approximately 1 g fresh weight were taken from each root system and stored in 15% ethanol. Roots were then stained with 0.5% trypan blue in lactic acid [30] and a minimum of 200 intersections per sample was counted using a modified gridline intersection method [31,32] and a dissecting microscope with 50× magnification.

### 2.3. Plant Material, Cultivation and Irrigation

Seeds of ‘HSD6719’, ‘HSD7058’ and ‘Sarah’ were kindly provided by the gene bank of the Agricultural Research Corporation, Wad Madani, Sudan. Seeds of ‘Khartoumia’ were obtained from a seed provider in the local market ‘SougBahri’, Khartoum, Sudan. The ‘Clemson Spineless’ seeds were obtained from Vertriebsgesellschaft Quedlinburger Saatgut mbH, Aschersleben, Germany. Three okra seeds were seeded per pot.

The substrate was watered with deionized water up to 20% (*w*/*w*). One week after germination, the seedlings were thinned to 1 seedling per pot considering plants of similar size in all pots. Starting 6 weeks after sowing, substrate water content was either maintained at 20% (+W) or reduced to 10% (*w*/*w*) (−W) to impose drought stress. Daily evapotranspiration from each planting pot was gravimetrically estimated by placing the pot on a balance and measuring the weight. The water lost from each pot was replaced daily with deionized water to maintain the average soil water content according to the respective treatment.

### 2.4. Harvest, Quantification of AMF Colonized Root Length and Plant Growth Measurement 

Plants were harvested 11 weeks after seeding. At harvest, shoots were separated directly above the surface of the substrate. The shoots were separated into leaves and stems and the fresh weight was recorded (for stems and leaves inclusive of lost leaves). Roots were carefully removed, washed of the growth substrate and the fresh weight was recorded. Two representative root samples each consisting of 1 g fresh weight were obtained by mixing 3 subsamples collected from the root system after dividing it into 3 vertical sections starting from the top of the root. Half of the representative root samples were stored in 15% ethanol to determine the AMF colonization rate (Section 2.2). The second half was scanned and analyzed with image processing software WinRHIZO Arabidopsis 2012b (Regent Instruments, Québec, Canada). Weight-based upscaling to the bulk root was implemented to estimate the mean root diameter and root length density. Leaf area (LA) was measured with the LI-3100 Area Meter (LICOR, Lincoln, NE, USA). The plant material (root, stem and leaves inclusive of lost leaves) was dried at 60 °C for 72 h in a drying oven, after which the plant dry weight (DW) was recorded. The mycorrhizal dependency was calculated within each treatment (cultivar × substrate water content) by using the individual values of the total dry biomass of +M plants and mean values of the total dry biomass of −M plants according to Bowles, Jackson and Cavagnaro [16] using the following formula:

%MD = (total DW (mycorrhizal plant) − mean total DW (non-mycorrhizal plants))/(mean total DW (non-mycorrhizal plants)) × 100

### 2.5. Nutrient Analysis and Statistics

Dried shoot material, including stem and leaves inclusive of lost leaves (subsample of 200–300 mg), were digested with 5 mL concentrated HNO_3_ (65%) and 2 mL H_2_O_2_ (30%) at 200 °C for 15 min in a microwave (MARSXpress 250/50; CEM Corporation, Charlotte, NC, USA), filtrated and taken up to 50 mL with distilled water. The concentrations of P, Fe and Zn were measured in the filtrate using an ICP-OES analyzer (Thermo Scientific iCAP™ 7400; Thermo Fisher, Taufkirchen, Germany) separate for element-specific wavelength according to the manufacturer’s instructions. To quantify N concentrations in the shoot, approximately 15 mg of the pulverized plant material, including stem and leaves inclusive of lost leaves, was analyzed after dry oxidation in an elemental analyzer (Elementar Vario EL, Elementar, Germany) following the Dumas method.

Data with a normal distribution (Kolmogorov-Smirnov test; *p* > 0.05) and homogeneity of variance (Levene’s test; *p* > 0.05) were subjected to factorial analysis of variance (ANOVA). The multiple comparison Tukey’s test was used to assign significant differences between means, applying a cut-off of *p* < 0.05. Data that were not normally distributed were subjected to the Kruskal-Wallis test (*p* < 0.05). Statistics were performed using the STATISTICA program version 12 (StatSoft Inc., Tulsa, OK, USA). 

## 3. Results

**Figure 1:**
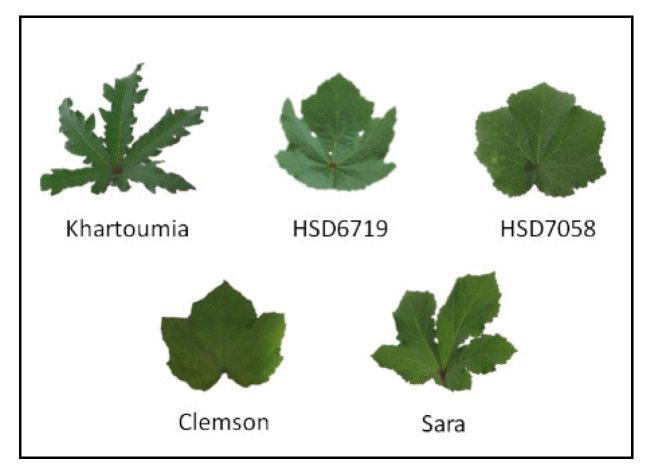
Leaf morphology of the five okra cultivars used in the experiment.

The five tested cultivars exhibited different leaf shapes (Figure 1).

### 3.1. Mycorrhizal Status

**Figure 2:**
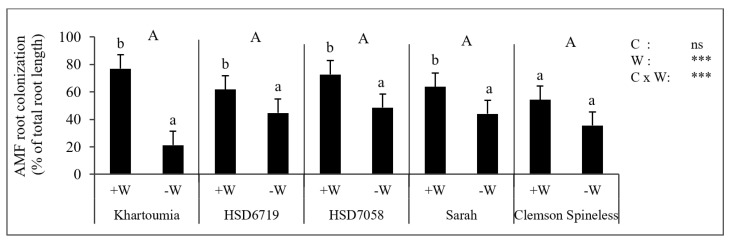
AMF colonized root length in percent of the total root length at the time of harvest of the five tested cultivars. Substrate water content was either maintained at 20% (+W) or reduced to 10% *w*/*w* (−W). Asterisks indicate significant differences in the cultivar (C), substrate water content (W) and their interaction (Kruskal-Wallis test, *n* = 4, *p* < 0.05 (*), *p* < 0.01 (**), *p* < 0.001 (***) and *p* > 0.05 (ns)). Shown are mean values ± standard deviation. Within each cultivar, the mean values followed by the same small letter are not significantly different (Tukey’s test, *n* = 4, *p* < 0.05). Cultivars followed by the same capital letter are not significantly different (Tukey’s test, *n* = 4, *p* < 0.05).

No colonization was observed in the roots of plants of the −M treatments. The percentage of colonized root length was not distinctly different among the cultivars. However, the drought stress treatment significantly decreased the root colonization rate in all cultivars except for Clemson Spineless, where the reduction was not significant (Figure 2).

### 3.2. Evapotranspiration

**Figure 3:**
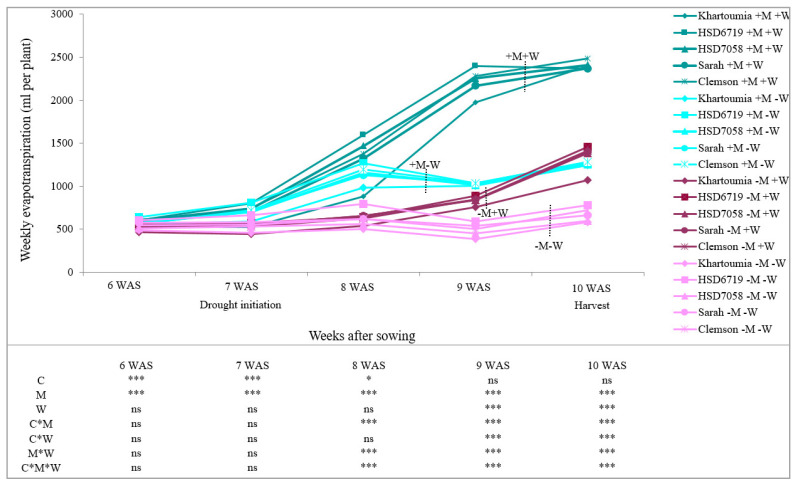
Weekly evapotranspiration (mL per plant). Plants were non-inoculated (−M) or inoculated (+M) with *R. irregulare*. Substrate water content was either maintained at 20% (+W) or reduced 10% *w*/*w* (−W). Asterisks indicate significant differences in cultivar (C), AM inoculation (M) and substrate water content (W) and their interactions (Kruskal-Wallis test, *n* = 4, *p* < 0.05 (*), *p* < 0.01 (**), *p* < 0.001 (***) and *p* > 0.05 (ns)). Results are shown as mean value ± standard deviation.

The effect of the okra cultivar on plant evapotranspiration rates was significant between 6 and 8 weeks after seeding (WAS). After that, the differences in evapotranspiration rates between cultivars levelled off and were not significant (Figure 3). Two weeks after initiating the drought stress treatment (9 WAS), evapotranspiration rates were significantly affected by drought stress. For instance, +W plants had higher evapotranspiration rates than −W plants in both +M and −M treatments (Figure 3). The evapotranspiration of +M plants was more extremely limited by drought treatment compared to −M plants (Figure 3).

### 3.3. Plant Shoot Height

**Figure A1:**
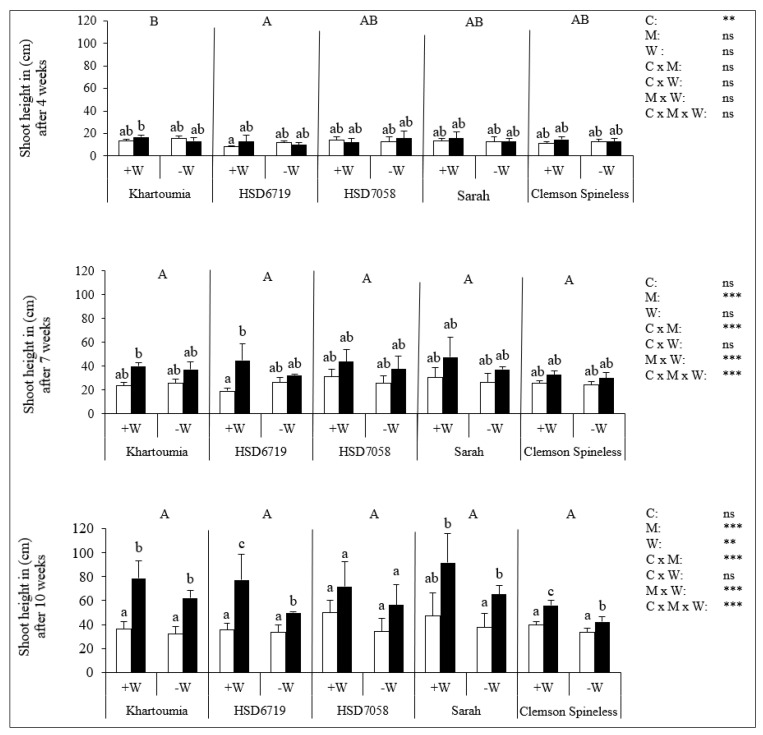
Shoot height (cm) of five okra cultivars measured 4, 7 and 10 weeks after seeding. Plants were non-inoculated (white bars) or inoculated with *R. irregulare* (black bars). Asterisks indicate significant differences of the factors cultivar (C), AM-inoculation (M) and substrate water content (W) and their interactions (Kruskal-Wallis test, *n* = 4, *p* < 0.05 (*), *p* < 0.01 (**), *p* < 0.001 (***) and *p* > 0.05 (ns)). Shown are mean values ± standard deviation. Within each cultivar mean values followed by the same small letter are not significantly different (Tukey test, *n* = 4, *p* < 0.05). Cultivars followed by the same capital letter are not significantly different (Tukey test, *n* = 4, *p* < 0.05).

At 4 WAS (first measurement), the plant shoot height was found to not be affected by the inoculation nor substrate water content but by the cultivar (Figure A1). At 7 WAS, shoot height was significantly affected by the inoculation treatment but not the cultivar or the substrate water content (Figure A1). At 10 WAS and 3 weeks after drought initiation (third measurement), the shoot height of okra plants was significantly lower in the drought stress treatment. Under drought stress, the shoot height was significantly higher in mycorrhizal plants compared to non-mycorrhizal plants in all the cultivars except HSD7058 (Figure A1). 

### 3.4. Shoot Dry Biomass 

**Figure 4:**
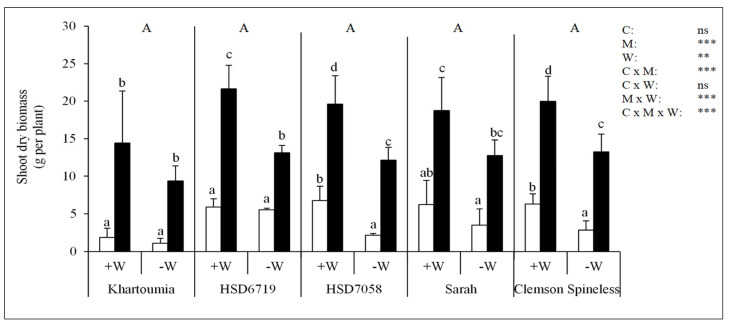
Shoot dry biomass for the five okra cultivars. Plants were non-inoculated (white bars) or inoculated with *R. irregulare* (black bars). Asterisks indicate significant differences in cultivar (C), AM inoculation (M) and substrate water content (W) and their interactions (Kruskal-Wallis test, *n* = 4, *p* < 0.05 (*), *p* < 0.01 (**), *p* < 0.001 (***) and *p* > 0.05 (ns)). Shown are mean values ± standard deviation. Within each cultivar mean values followed by the same small letter are not significantly different (Tukey’s test, *n* = 4, *p* < 0.05). Cultivars followed by the same capital letter are not significantly different (Tukey’s test, *n* = 4, *p* < 0.05).

Drought stress significantly affected the shoot dry biomass (−M/+W vs. −M/−W). The shoot dry biomass was significantly lower in −W plants compared to +W plants for HSD7058 and Clemson Spineless (69% and 56% decrease in the shoot DB, in the respective cultivars). The decrease was slightly lower for Khartoumia, HSD6719 and Sarah (42%, 5% and 44% decrease in the shoot DB, in the respective cultivars) (Figure 4). The shoot dry biomass did not differ significantly among the cultivars but was significantly affected by the inoculation treatment. AMF colonization significantly increased the shoot dry biomass in all cultivars under both +W and −W treatments (Figure 4).

### 3.5. Leaf Area and Leaf-to-Stem Ratio

In comparing the five cultivars, the leaf area from lowest to highest was Khartoumia < Sarah < HSD7619, HSD7058 and Clemson Spineless. Drought stress (−M/+W vs. −M/−W) decreased the leaf area only in HSD7058 irrespective of the inoculation treatment. AM inoculation increased the leaf area remarkably in comparison to the non-AM treatment, irrespective of the water treatment or cultivar (Table A2). The leaf-to-stem dry weight ratio was statistically similar among the tested cultivars (Table A2). Drought stress resulted in significantly lower leaf–stem ratios compared to well-watered conditions (−M/+W vs. −M/−W) in HSD6719, Sarah and Clemson Spineless (Table A2). AM-inoculated plants had a lower leaf–stem ratio in HSD6719 compared to non-inoculated plants, while other cultivars remained unaffected by the inoculation treatment (Table A2).

### 3.6. Root Dry Biomass 

**Figure 5:**
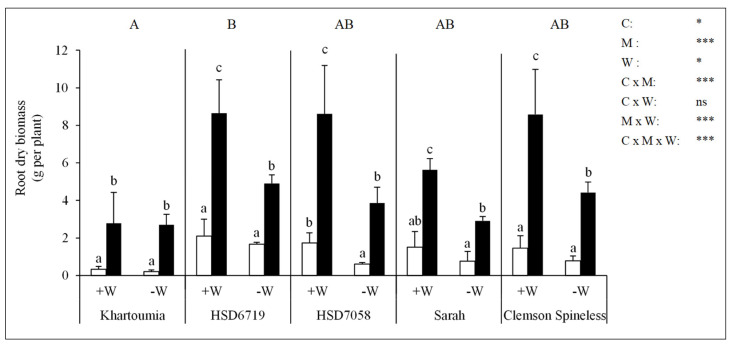
Root dry biomass in five okra cultivars. For definitions of treatments and statistics, refer to the text for Figure 4.

The root dry biomass differed significantly according to the cultivar. HSD6719 had the highest root dry biomass while Khartoumia had the lowest (Figure 5). Drought stress decreased the root dry biomass in comparison to ample water conditions (−M/+W vs. −M/−W). This decrease was significant in HSD7058 (Figure 5). AMF colonization (+M vs. −M) significantly increased the root dry biomass of okra plants irrespective of cultivar or substrate water content (Figure 5).

### 3.7. Root Development

In general, the values of specific root length (SRL) in cm/g root DW and the root length density (RLD) in cm/cm^3^ were remarkably high especially in +M plants as compared to values published for okra plants. The root diameter was comparable to that found in published studies [33,34,35,36,37].

**Figure 6:**
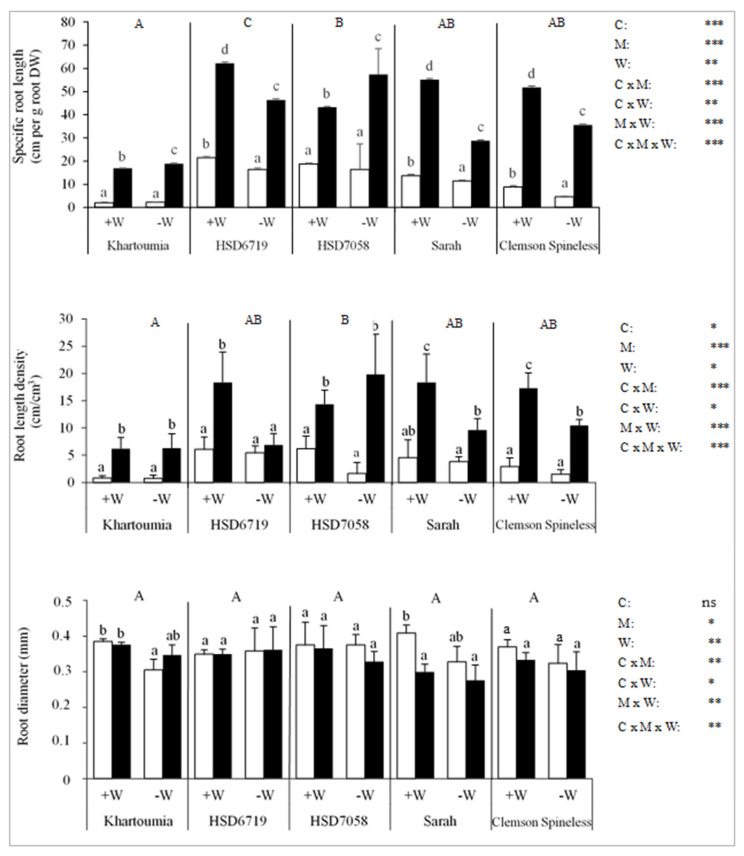
Specific root length (**upper**), root length density (**middle**) and root diameter (**lower**) in the five okra cultivars. For definitions of treatments and statistics, refer to the text for Figure 4.

The SRL differed significantly between the cultivars and was in the following order from lowest to highest: Khartoumia < Sarah and Clemson Spineless HSD7058 < HSD6719 (Figure 6). Drought stress significantly reduced SRL in Clemson Spineless, Sarah and HSD6719 irrespective of the inoculation treatment, while Khartoumia and HSD7058 remained unaffected by drought stress (Figure 6). Inoculation with AMF significantly increased the specific root length in all treatments (Figure 6). 

The RLD varied significantly depending on the cultivar and was in the following order from lowest to highest: Khartoumia < HSD6719, Sarah and Clemson Spineless < HSD7058. The RLD of non-mycorrhizal plants was not affected by the drought stress, irrespective of the cultivar (Figure 6). However, among mycorrhizal plants, drought stress significantly reduced the RLD of HSD6719, Sarah and Clemson Spineless compared to well-watered plants. Inoculation with AMF significantly increased the RLD under both +W and −W treatments in all cultivars except for HSD7619, where a significant effect of AMF inoculation was observed only in +W treatment. 

The root diameter was statistically similar for all five studied cultivars. Drought stress decreased the root diameter only in Khartoumia in treatment −M/−W compared with the well-watered counterpart. AM-inoculated plants showed significantly smaller root diameters than non-inoculated plants in the case of Sarah at +W but not for any other cultivar (Figure 6).

The root-to-shoot dry weight ratio differed significantly according to the cultivar in the following order: Khartoumia < Sarah < HSD7058 and Clemson Spineless < HSD6719. Drought stress treatment did not affect the root–shoot ratio except for Khartoumia, where +M/+W plants showed a lower root–shoot ratio compared to +M/−W plants. AM inoculation (+M vs. −M) resulted in significantly higher root–shoot ratios for HSD7058 and Clemson Spineless at +W treatments, and a similar effect was shown by trend at the corresponding −W treatments (Table 1).

### 3.8. The Mycorrhizal Dependency (MD)

**Figure 7:**
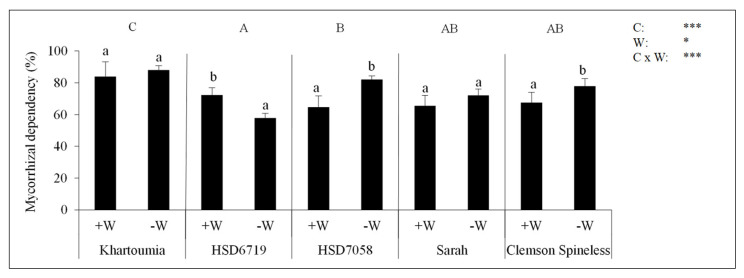
Mycorrhizal dependency in percent calculated for the five okra cultivars. The mycorrhizal dependency was calculated within each treatment (C × W) by using the individual values of the total dry biomass of +M plants and mean values of the total dry biomass of −M plants. It was calculated according to the formula: %MD = (total DW (mycorrhizal plant)—mean total DW (non-mycorrhizal plants))/(mean total DW (non-mycorrhizal plants)) × 100. For definitions of treatments and statistics, refer to the text for Figure 2.

The five cultivars differed significantly in the degree to which they depended on AMF to produce total biomass. The order from highest to lowest MD was Khartoumia > HSD7058 > Sarah and Clemson Spineless > HSD6719. The improved cultivar Khartoumia showed higher MD by 15%, 46%, 17% and 32% compared with Clemson Spineless, the wild-type Sarah and the HSD7058 and HSD6719 landraces, respectively. Under drought stress treatment HSD7058 and Clemson Spineless had a significantly higher MD (by 27% and 15%, respectively) compared to ample water treatment. By contrast, HSD6719 showed a reverse trend with a significantly lower MD (by 20%) in the −W than in +W treatment (Figure 7).

### 3.9. Plant Nutrient Status

The shoot P concentration ranged between 0.6 and 1.9 mg g^−1^ DW across all the treatments and cultivars (Table 2). Significantly lower shoot P concentrations were present in −W compared to +W treatment in HSD7619 (−M treatment) as well as HSD7619, HSD7058 and Sarah (+M treatments). Compared to non-inoculated plants, AM inoculation increased shoot P concentration in Khartoumia, HSD6719, HSD7058, Sarah and Clemson Spineless by 58%, 83%, 79%, 89% and 118%, respectively, when plants were well-watered, and by 58%, 100%, 70%, 54% and 100%, respectively, when they were drought-stressed (Table 2).

Shoot N concentration ranged between 13 and 43 mg g^−1^ DW across all treatments and cultivars (Table 2). Treatments with low shoot biomass had higher shoot N concentrations, i.e., −M vs. +M plants (all cultivars) and −W vs. +W (HSD7058) (Table 2). 

Shoot Fe concentration ranged between 46 and 148 mg kg^−1^ DW (Table 2). Fe concentration was not significantly affected by drought stress or cultivar. Higher shoot Fe concentrations were linked to lower shoot biomass, i.e., higher Fe concentrations in −M than +M plants in HSD7619, HSD7058 and Clemson Spineless (Table 2).

Shoot Zn concentrations ranged between 35 and 47 mg kg^−1^ DW and were not significantly affected by drought stress except in Clemson Spineless, where shoot Zn concentration was lower at −W than at +W, irrespective of AM inoculation (Table 2). AMF inoculation did not affect the concentration of Zn in the shoot of any plants in all treatments (Table 3 and Table 4). 

The total content of P, N, Zn and Fe in the shoot was in accordance with the differences in shoot biomass in the respective treatments, significantly higher in +W treatments as compared to −W and higher in +M as compared to −M.

## 4. Discussion

In this study, we investigated the effect of AMF inoculation on evapotranspiration, mineral nutrient uptake and shoot and root development in five cultivars of okra under ample water and drought stress conditions. 

As anticipated, the five showed different phenotypes, i.e., differences in leaf shape and area, root dry biomass, biomass allocation (root–shoot ratio), SRL and RLD. Previous studies also described variations in okra total dry biomass, leaf area and root length when different accessions or genotypes were grown with equal access to resources [36,37,38,39]. 

In the present study, exposure to drought stress reduced okra plant shoot dry biomass in all five accessions that were tested, but HSD7058 and Clemson Spineless were the most affected. 

Compared with standard values cited by Hochmuth et al. [40], concentrations of P in the shoot tissue of control plants (−M) were generally indicative of severe P deficiency when plants were drought-stressed (irrespective of cultivar). Despite the higher P concentrations in the shoot of −M/+W plants as compared to −M/−W plants, shoot P concentrations of +W plants were also indicative of P deficiency. This result indicates that under the current experimental conditions, P availability was not constrained by drought treatment only, but also by the chemical substrate conditions. Indeed, calcareous soils such as the substrate used in this experiment usually tend to immobilize soluble P to a high degree [5]. Levels of N in the shoots were within the recommended average for adequate plant growth [40] in all treatments except for +M/+W treatment. The below-average N concentrations in +M/+W plants could be attributed to their higher biomass compared to the other treatments. Accordingly, the high biomass led to N dilution in the shoot tissues. A similar effect was also observed for Fe, where its concentrations in the shoot tissue of the relatively higher biomass plants were generally indicative of deficiency. The concentrations of Zn in the shoot tissues were sufficiently high in all treatments.

The contribution of AMF to plant P uptake (total content of P in the shoot) was reported in soils where low P availability was induced by drought. Moreover, it was also reported in soils with a low total P content or with a high P fixing capacity [41,42,43]. Other studies reported that under depleted soil moisture, AMF inoculation resulted in higher shoot N and K concentrations compared to non-inoculated counterparts [44,45]. Here, unlike concentrations of N and Fe that were lower in +M plants compared to −M plants due to the dilution effect, concentrations of P were higher or similar in +M compared to −M plants. This result doubtlessly indicated the significant contribution of AMF to P uptake, which kept pace with the rapid growth of +M plants compared to −M plants. Moreover, the contribution of AMF to P uptake occurred not only when P was limited by drought but also under well-watered conditions.

Different combinations of plant or AM fungal species have shown different responses in terms of P uptake and total biomass accumulation [46]. Previous studies showed that plant inoculation with *Rhizoglomus irregulare* resulted in increased shoot total content of P and total dry biomass accumulation in different plant species, such as *Abelmoschus esculentus*, *Medicago truncatula*, *Medicago sativa*, *Capsicum annuum* and *Sorghum bicolor* [47,48,49,50]. However, the increase in the shoot total content of P and total dry biomass accumulation varied depending on the plant species. Other studies discussed the role of the AM fungal species in the output of the symbiosis in terms of shoot and root total content of P and total dry biomass accumulation. For instance, the inoculation of the okra plant with *Rhizoglomus irregulare* and *Acaulospora Laevis* resulted in remarkably different shoot and root total P content and total dry biomass depending on the AMF species [47]. Our data showed a significant interaction between the AMF inoculation and the cultivar. Our study reveals that besides the plant and the AMF species factors, the cultivar plays a role in the output of okra-AMF symbiosis in terms of P uptake. 

Under drought stress conditions, one of the most common explanations for the enhanced P status in AM-inoculated plants was the increased absorbing surface provided by AMF hyphae, which conferred the ability to explore soil pores that retain nutrients in the soil solution as the soil dries [51,52]. In our experiment, increased RLD from AMF inoculation may indicate that the net effect of AMF inoculation on RLD was complementary to the AMF pathway (the hyphal pathway) for enhancing P uptake, especially under limited P conditions. Our results are in contrast to previous findings in safflower and wheat, where RLD remained unchanged upon AMF inoculation with *Glomus etunicatum* when plants were water-stressed [53]. The results are also in contrast with Bitterlich et al. [54], who found that the RLD of tomato plants was not affected by inoculation with *F. mosseae* when plants were exposed to sequential drying episodes. We speculate that plant species with a high MD, such as okra, may show root morphological responses to AMF colonization different from that of plant species with lower MD, such as most grasses or tomatoes. The higher values of SRL and RLD in our study compared to previously published values [33,34,35] could be attributed to the age of the plant at the time of RLD measurement. We measured the root parameters 3 months after cultivation while the other studies measured the parameters 1–2 months after cultivation.

Evapotranspiration data showed that under −W conditions, much lower daily evapotranspiration was observed in −M and +M treatments as compared to their +W counterparts (irrespective of cultivar). However, under −W treatment, +M plants had higher evapotranspiration rates in comparison with −M plants. These findings confirm earlier studies where increased transpiration for AM compared with non-AM plants was observed under conditions of drought [55,56]. The higher evapotranspiration rates under drought stress conditions in +M plants compared to −M plants could be attributed to the improved P nutrition (concentration and total content in the shoot tissues) due to AMF inoculation leading, for example, to larger leaves (as shown in Table A2) or more stomatal openings. This result is not consistent with an earlier study in *Rosa hybrida* [57], which reported that the enhancement of P nutrition by AMF was not correlated with transpiration rates. 

The contribution of AMF to okra plant P uptake was sufficiently high to support adequate plant growth even when P was limited in the substrate, as AMF significantly enhanced the okra plant total dry biomass (presented as shoot and root dry biomass) of the five cultivars not only under −W but also under +W conditions. However, the mycorrhizal dependency (MD), or the degree to which okra plants depended on AMF in producing total dry biomass, varied among the cultivars. Among the five cultivars, HSD6719 had the lowest MD, while Khartoumia showed the highest. HSD6719 had the strongest root system in terms of higher root biomass, root–shoot ratio, SRL, whereas Khartoumia had the weakest root system. It was reported that lower mycorrhiza-dependent plants have finer roots (higher length per unit root dry weight) [15]. Here, HSD6719 showed the highest SRL among the cultivars, which probably confers the plants with a better capability to forage soil for P, especially under drought stress [58]. The rate of shoot growth is one important physiological factor that determines the rate of nutrient demand [59]. We observed that the cultivar with the highest MD (Khartoumia) had a lower root–shoot ratio compared to HSD6719, which had the lowest MD value. The root–shoot ratio data indicate that Khartoumia probably had a higher nutrient demand and consequently higher MD compared to HSD6719. Compared to ample water conditions, drought stress induced higher MD. Interestingly, this was observed in HSD7058 and Clemson Spineless, which showed sensitivity to drought stress (reduced shoot dry biomass in −M/+W compared to −M/+W). 

In addition to the physiological traits, our data suggest a possible relationship between the breeding background of the cultivars and their MD. Some authors have discussed the genetic relationship of MD in genotypes within a species. For instance, it has been reported that high MD is more likely for wild-types and in older accessions and cultivars that pre-date the heavy fertilization era, while lesser MD can be expected in modern cultivars. It is believed that improved cultivars were selected for breeding programs under high P fertilizer conditions that reduced the frequency of genes for mycorrhizal dependence [60]. For example, an unimproved soybean cultivar was found to have higher MD for total dry biomass compared to an improved cultivar [20]. On the other hand, contrasting results have also been reported, where modern cowpea cultivars showed a higher MD in total dry biomass and shoot total content of P and N than the wild-type [22]. In the present experiment, the breeding-improved Khartoumia cultivar showed a higher MD compared to wild-type Sarah and the farmer’s local cultivars (landraces) HSD6719 and HSD7058. However, the introduced cultivar Clemson Spineless showed a similar MD to the wild-type and the local farmer cultivars. Wild okra grows in natural sites, whereas landraces are grown by subsistence farmers under low-input agriculture. Thus, these cultivars are expected to be more adapted to low P conditions, which was reflected in the lower MD for P uptake compared to the breeding-improved Khartoumia. Our results indicate that modern plant breeding programs do not necessarily lead to the suppression of plant MD. Under field conditions, soil microbiota interactions exist unlike in pot experiments under controlled conditions and sterilized soil that are often used in mycorrhizal experiments. Therefore, despite the positive results of our study, field experiments are needed to confirm these results. 

## 5. Conclusions

The results of our study indicate the remarkable potential of AMF to enhance both P uptake, in terms of total shoot P content, and total dry biomass of okra plant species not only under ample water conditions but also under drought stress that induces strong limitation regarding P availability. 

We revealed new findings regarding the role of the cultivar in the outcome of okra-AMF symbiosis, especially under limited P and soil moisture conditions. 

Our study indicates the significance of the presence of AMF propagules in field soil for increasing yield production of high MD cultivars such as Khartoumia or drought-affected cultivars such as HSD7058 and Clemson Spineless. To translate the output of this research to field conditions, further experiments on a larger okra cultivar set are needed. 

## Figures and Tables

**Table 1:** Ratio of root–shoot (based on DW) of the five okra cultivars. Plants were non-inoculated (−M) or inoculated (+M) with *R. irregulare*. Substrate water content was either maintained at 20% (+W) or 10% *w*/*w* (−W). For definitions of treatments and statistics, refer to the text for Figure 3. Shown are mean values ± standard deviation. Within each cultivar mean values followed by the same small letter are not significantly different (Tukey’s test, *n* = 4, *p* < 0.05). Cultivars followed by the same capital letter are not significantly different (Tukey’s test, *n* = 4, *p* < 0.05).

Khartoumia				HSD6719				HSD7058				Sarah				Clemson Spineless			
+W		−W		+W		−W		+W		−W		+W		−W		+W		−W	
−M	+M	−M	+M	−M	+M	−M	+M	−M	+M	−M	+M	−M	+M	−M	+M	−M	+M	−M	+M
**Ratio root–shoot**																			
0.19 a ± 0.04	0.19 a ± 0.06	0.20 ab ± 0.04	0.29 b ± 0.03	0.34 a ± 0.11	0.39 a ± 0.04	0.30 a ± 0.01	0.37 a ± 0.03	0.27 a ± 0.10	0.43 b ± 0.08	0.28 ab ± 0.01	0.32 ab ± 0.09	0.24 ab ± 0.04	0.31 b ± 0.07	0.21 a ± 0.03	0.23 ab ± 0.03	0.22 a ± 0.06	0.43 b ± 0.09	0.29 ab ± 0.10	0.35 ab ± 0.12
A				C				BC				AB				BC			
C:				***															
M:				***															
W:				ns															
C × M:				ns															
C × W:				ns															
M × W:				ns															
C × M × W:				ns															

**Table 2:** Shoot concentration of P, N, Fe and Zn of five okra cultivars. Plants were non-inoculated (−M) or inoculated (+M) with *R. irregulare*. Substrate water content was either maintained at 20% (+W) or 10% *w*/*w* (−W). For statistics, refer to the text for Figure 3. Shown are mean values ± standard deviation. Within each cultivar mean values followed by the same small letter are not significantly different (Tukey’s test, *n* = 4, *p* < 0.05). Cultivars followed by the same capital letter are not significantly different (Tukey’s test, *n* = 4, *p* < 0.05).

Khartoumia				HSD6719				HSD7058				Sarah				Clemson Spineless			
+W		−W		+W		− W		+W		−W		+W		−W		+W		−W	
−M	+M	−M	+M	−M	+M	−M	+M	−M	+M	−M	+M	−M	+M	−M	+M	−M	+M	−M	+M
**P concentration (mg g^−1^ DW)**																			
0.90 ab ± 0.17	1.43 b ± 0.42	0.65 a ± 0.06	1.03 ab ± 0.15	0.90 b ± 0.08	1.65 d ± 0.21	0.60 a ± 0.08	1.20 c ± 0.08	0.98 ab ± 0.05	1.75 c ± 0.19	0.63 a ± 0.06	1.08 b ± 0.21	0.88 ab ± 0.05	1.65 c ± 0.25	0.60 a ± 0.12	0.93 b ± 0.10	0.85 ab ± 0.06	1.85 b ± 0.19	0.60 a ± 0.20	1.20 ab ± 0.00
A				A				A				A				A			
**N concentration (mg g^−1^ DW)**																			
33.15 bc ± 2.47	17.83 a ± 6.31	42.97 c ± 3.08	25.08 ab ± 4.28	33.88 b ± 4.51	12.89 a ± 1.39	34.64 b ± 2.53	17.52 a ± 1.02	30.46 c ± 1.60	13.64 a ± 1.78	34.20 c ± 2.30	18.64 b ± 1.74	27.38 b ± 4.56	13.74 a ± 2.65	33.13 b ± 3.43	18.60 a ± 2.03	30.45 b ± 3.50	12.96 a ± 0.87	27.27 ab ± 10.19	17.38 ab ± 1.74
A				A				A				A				A			
**Fe concentration (mg kg^−1^ DW)**																			
84.79 ab ± 22.71	48.12 a ± 15.14	124.06 b ± 81.19	58.18 ab ± 14.29	125.10 b ± 53.20	56.18 a ± 9.68	74.66 ab ± 11.59	74.57 ab ± 21.48	102.57 b ± 14.28	58.51 a ± 24.93	76.80 ab ± 26.81	43.18 a ± 2.90	92.36 ab ± 31.19	46.35 a ± 15.38	128.01 b ± 107.00	65.28 ab ± 20.15	148.25 b ± 107.40	53.87 a ± 13.18	75.24 ab ± 41.34	61.59 ab ± 20.43
A				A				A				A				A			
**Zn concentration (mg kg^−1^ DW)**																			
37.34 a ± 4.58	38.43 a ± 6.55	43.50 a ± 13.82	39.42 a ± 3.56	47.44 a ± 3.43	44.44 a ± 6.02	38.29 a ± 6.38	39.42 a ± 2.62	48.42 b ± 4.44	47.06 ab ± 6.81	43.77 ab ± 6.43	35.96 a ± 5.28	42.98 a ± 3.27	43.71 a ± 6.70	40.00 a ± 5.57	35.20 a ± 7.37	47.43 bc ± 6.66	52.66 c ± 2.96	35.87 a ± 3.77	39.49 ab ± 4.06
A				A				A				A				A			

**Table 3:** Shoot total content of P, N, Fe and Zn of five okra cultivars. Plants were non-inoculated (−M) or inoculated (+M) with *R. irregulare*. Substrate water content was either maintained at 20% (+W) or 10% *w*/*w* (−W). For definitions of treatments and statistics, refer to the text for Figure 3. Shown are mean values ± standard deviation. Within each cultivar mean values followed by the same small letter are not significantly different (Tukey’s test, *n* = 4, *p* < 0.05). Cultivars followed by the same capital letter are not significantly different (Tukey’s test, *n* = 4, *p* < 0.05).

Khartoumia				HSD6719				HSD7058				Sarah				Clemson Spineless			
+W		−W		+W		− W		+W		−W		+W		−W		+W		−W	
−M	+M	−M	+M	−M	+M	−M	+M	−M	+M	−M	+M	−M	+M	−M	+M	−M	+M	−M	+M
**P total content (mg per plant)**																			
1.60 a ± 0.88	22.20 b ± 13.40	0.71 a ± 0.45	9.46 b ± 1.55	5.23 b ± 0.66	35.28 d ± 3.69	3.30 a ± 0.35	15.69 c ± 0.57	6.50 b ± 1.52	33.80 d ± 3.21	1.37 a ± 0.27	13.10 c ± 3.21	5.44 ab ± 2.82	30.82 c ± 7.77	2.22 a ± 1.66	11.86 b ± 2.79	5.33 b ± 1.00	36.79 d ± 6.46	1.58 a ± 0.46	15.88 c ± 2.84
A				A				A				A				A			
**N total content (mg per plant)**																			
62.24 a ± 42.15	242.17 b ± 96.82	45.11 a ± 24.29	229.06 b ± 16.92	197.36 a ± 37.63	275.42 b ± 15.11	190.96 a ± 14.29	229.36 ab ± 12.12	203.46 b ± 48.74	263.11 b ± 27.32	73.73 a ± 12.78	224.34 b ± 12.24	162.53 ab ± 80.93	251.67 b ± 38.23	112.55 a ± 60.59	234.15 b ± 12.61	188.71 b ± 22.12	257.05 b ± 30.82	82.65 b ± 49.72	28.47 a ± 37.48
A				A				A				A				A			
**Fe total content (mg per plant)**																			
175.6 ab ± 131.5	635.7 c ± 251.0	1074 a ± 41.09	532.2 bc ± 127.5	753.3 ab ± 392.34	1200 b ± 183.33	410.3 a ± 53.08	983.7 b ± 311.9	686.8 ab ± 185.70	1116.7 b ± 394.22	157.0 a ± 61.97	523.3 a ± 68.45	623.1 a ± 403.31	859.4 a ± 288.99	336.0 a ± 141.5	814.4 a ± 201.7	909.3 ab ± 594.44	1084.9 b ± 357.02	236.1 a ± 208.41	812.8 ab ± 307.23
A				A				A				A				A			
**Zn total content (mg per plant)**																			
87.1 ab ± 67.00	676.2 b ± 338.02	51.1 a ± 26.81	470.5 ab ± 72.76	373.7 a ± 72.62	1324.3 c ± 105.03	126.8 a ± 37.99	710.3 b ± 62.81	415.2 b ± 131.49	1296.8 c ± 98.53	273.6 a ± 31.87	577.1 b ± 118.5	335.0 ab ± 177.48	1059.0 c ± 193.67	174.0 a ± 107.9	544.0 b ± 81.0	359.0 a ± 46.14	1502.0 c ± 271.26	132.9 a ± 64.42	698.1 b 114.34
A				A				A				A				A			

**Table 4:** Significant differences in cultivar (C), AM inoculation (M) and substrate water content (W) and their interactions are indicated with asterisks (Kruskal-Wallis test, *n* = 4, *p* < 0.05 (*), *p* < 0.01 (**), *p* < 0.001 (***) and *p* > 0.05 (ns)).

	P Concentration	N Concentration	Fe Concentration	Zn Concentration	P Content	N Content	Fe Content	Zn Content
C:	ns	ns	ns	ns	ns	ns	ns	ns
M:	***	***	***	ns	***	***	***	***
W:	***	*	ns	***	***	**	**	***
C × M:	***	***	***	ns	***	***	***	***
C × W:	*	ns	ns	**	ns	ns	**	ns
M × W:	***	***	***	ns	***	***	***	***
C × M × W:	***	***	***	ns	***	***	ns	***

**Figure A2:**
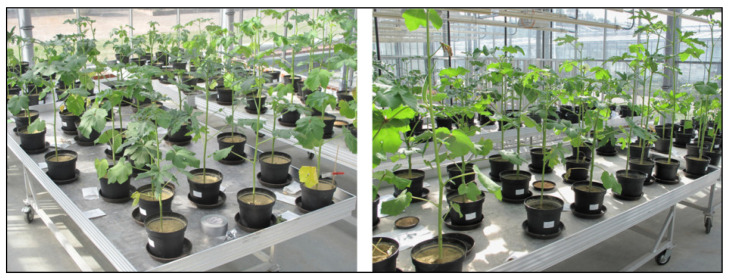
Greenhouse experiment (okra plants in a randomized design on tables inside the greenhouse).

**Figure A3:**
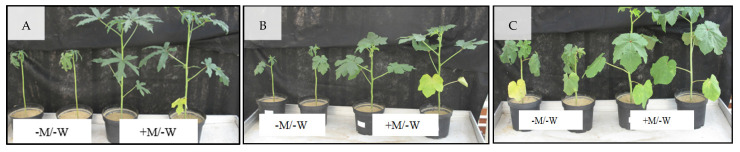
Mycorrhizal (+M) and non-mycorrhizal (−M) okra plants grown under drought stress conditions (−W). Shown are cultivars Khartoumia, HSD6719 and Clemson Spineless ((**A**–**C**), respectively) on the day before harvest.

## Data Availability

Data supporting reported results can be requested from the corresponding author.

## Appendix A

**Table A1:** Properties of the mixture substrate.

Organic Matter Content (% of Substrate DW)	1.8
pH in (CaCl_2_)	7.5
Total cation exchange capacity (CEC) (µmol/g^−1^ DS)	66
CaCl_2_-extractable Mg (mg kg−^1^ DS)	78
CAL-extractable P (mg kg^−1^ DS)	6.9
CAL-extractable K (mg kg^−1^ DS)	36
Ca (mg kg^−1^ DS)	3000
CAT-extractable micronutrients:	
B (mg kg^−1^ DS)	0.04
Cu (mg kg^−1^ DS)	0.5
Mn (mg kg^−1^ DS)	12
Zn (mg kg^−1^ DS)	0.2

CAL-Extraction according to Schüller [61], CAT-Extraction according to Alt and Peters [62].

**Table A2:** Leaf area (in cm^2^ per plant) and ratio of leaf:stem (based on DW) of the five okra cultivars. Plants were non-inoculated (−M) or inoculated (+M) with *R. irregulare.* Substrate water content was either maintained at 20% (+W) or 10% *w*/*w* (−W). For statistics, refer to the text for Figure A1.

Khartoumia				HSD6719				HSD7058				Sarah				Clemson Spineless			
+W		−W		+W		− W		+W		−W		+W		−W		+W		−W	
−M	+M	−M	+M	−M	+M	−M	+M	−M	+M	−M	+M	−M	+M	−M	+M	−M	+M	−M	+M
**Leaf area (cm^2^ per plant)**																			
35.8 a ± 10.74	88.1 b ± 33.97	19.4 a ± 8.66	82.1 b ± 9.89	81.4 a ± 25.91	160.4 b ± 15.41	72.1 a ± 12.19	130.9 b ± 13.47	109.5 b ± 13.43	177.0 c ± 29.29	38.50 a ± 4.50	118.5 b ± 31.27	69.2 a ± 23.07	111.8 b ± 14.79	46.6 a ± 26.59	101.0 b ± 14.40	93.2 ab ± 13.05	35.8 a ± 10.74	88.1 b ± 33.97	19.4 a ± 8.66
A				C				C				B				C			
**Ratio leaf-to-stem**																			
1.11 a ± 0.19	0.69 a ± 0.22	0.88 a ± 0.25	0.94 a ± 0.13	1.91 b ± 0.15	0.84 a ± 0.24	1.02 a ± 0.27	1.14 a ± 0.42	1.15 a ± 0.46	1.43 a ± 0.14	0.71 a ± 0.75	0.85 a ± 0.24	1.31 b ± 0.65	0.78 ab ± 0.27	0.58 a ± 0.08	0.64 ab ± 0.08	1.40 b ± 0.37	1.11 a ± 0.19	0.69 a ± 0.22	0.88 a ± 0.25
A				A				A				A				A			
				Leaf area		Ratio leaf-to-stem													
C:				***		ns													
M:				***		ns													
W:				***		**													
C × M:				ns		ns													
C × W:				**		*													
M × W:				ns		**													
C × M × W:				ns		**													
